# Decreased Plasma Brain-Derived Neurotrophic Factor and Vascular Endothelial Growth Factor Concentrations during Military Training

**DOI:** 10.1371/journal.pone.0089455

**Published:** 2014-02-24

**Authors:** Go Suzuki, Shinichi Tokuno, Masashi Nibuya, Toru Ishida, Tetsuo Yamamoto, Yasuo Mukai, Keiji Mitani, Gentaro Tsumatori, Daniel Scott, Kunio Shimizu

**Affiliations:** 1 Military Medicine Research Unit, Test and Evaluation Command, Japan Ground Self Defense Force, Setagaya, Setagaya, Tokyo, Japan; 2 Department of Psychiatry, Japan Self-Defense Force Central Hospital, Setagaya, Setagaya, Tokyo, Japan; 3 Department of Defense Medicine, National Defense Medical College, Tokorozawa, Saitama, Japan; 4 Department of Psychiatry, National Defense Medical College, Tokorozawa, Saitama, Japan; 5 Department of Internal medicine, Japan Self-Defense Force Central Hospital, Setagaya, Setagaya, Tokyo, Japan; 6 Division of Molecular Psychiatry, Department of Psychiatry, Yale University School of Medicine, New Haven, Connecticut, United States of America; 7 Division of Behavioral Sciences, National Defense Medical College Research Institute, Tokorozawa, Saitama, Japan; Nathan Kline Institute and New York University School of Medicine, United States of America

## Abstract

Decreased concentrations of plasma brain-derived neurotrophic factor (BDNF) and serum BDNF have been proposed to be a state marker of depression and a biological indicator of loaded psychosocial stress. Stress evaluations of participants in military mission are critically important and appropriate objective biological parameters that evaluate stress are needed. In military circumstances, there are several problems to adopt plasma BDNF concentration as a stress biomarker. First, in addition to psychosocial stress, military missions inevitably involve physical exercise that increases plasma BDNF concentrations. Second, most participants in the mission do not have adequate quality or quantity of sleep, and sleep deprivation has also been reported to increase plasma BDNF concentration. We evaluated plasma BDNF concentrations in 52 participants on a 9-week military mission. The present study revealed that plasma BDNF concentration significantly decreased despite elevated serum enzymes that escaped from muscle and decreased quantity and quality of sleep, as detected by a wearable watch-type sensor. In addition, we observed a significant decrease in plasma vascular endothelial growth factor (VEGF) during the mission. VEGF is also neurotrophic and its expression in the brain has been reported to be up-regulated by antidepressive treatments and down-regulated by stress. This is the first report of decreased plasma VEGF concentrations by stress. We conclude that decreased plasma concentrations of neurotrophins can be candidates for mental stress indicators in actual stressful environments that include physical exercise and limited sleep.

## Introduction

The loaded stress levels of each participant in an occupational environment are variable, and precise stress management for an individual is important. Although self-questionnaires are effective tools to assess the current stress status, they are sometimes not effective because they are chiefly based on subjective evaluation. To support and validate questionnaire results, biomarkers that reflect the severity of mental stress are needed. In the present study, we examined plasma brain-derived neurotrophic factor (BDNF) and plasma vascular endothelial growth factor (VEGF) concentrations before, during, and after military training, and compared them with levels of self-evaluated stress, fatigue levels, and other biological parameters of muscle-escaping enzymes and sleep.

Decreased BDNF expression induced by stress was first found in the hippocampus in animal experiments [1], [2]. Although serum BDNF is mostly derived from platelets in coagulated clots, plasma BDNF is likely derived from vascular endothelial cells and the brain [3], [4]. BDNF protein molecules can cross the blood brain barrier bi-directionally via a high capacity and saturable transporter system [5]. Decreased plasma [6], [7] and serum [8], [9] BDNF concentrations have been observed in patients with depression and stressed subjects.

In contrast, physical exercise increases brain BDNF expression [10] and plasma BDNF concentration in young healthy men [11] and patients with amnestic mild cognitive impairment [12]. In addition, sleep deprivation, which is sometimes used in depression therapy, has been reported to increase serum BDNF concentration [13]. However, a pilot study with a small sample size did not reveal any alterations in plasma BDNF concentrations in severely depressed patients after sleep deprivation, despite a significant amelioration of depressive symptoms [14]. Therefore, we conducted the current study to examine whether plasma BDNF concentrations can be a candidate biological stress marker in subjects experiencing both psychological stress and significant physical exercise while simultaneously receiving a limited quantity and quality of sleep.

Compared to the relatively consistent results regarding the relationship between decreased blood BDNF concentrations and depression or stress, conflicting results have been reported for blood VEGF concentrations in patients with major depressive disorder [15], [16]. At present, an insufficient number of studies has been done, resulting in sparse reports of no alteration of blood VEGF concentration in patients with depression [17] as well as elevation of VEGF concentration upon treatment with antidepressants [18]. Furthermore, no reports have examined the blood VEGF levels induced by stress in normal healthy subjects. VEGF, as well as BDNF, have neuroplastic, neuroprotective, and neurogenic profiles in the brain [19], [20]. VEGF in the brain is up-regulated by various antidepressive treatments and down-regulated by stress, as been demonstrated previously in the hippocampus [21].

Subjective mental stress and physical fatigue were evaluated using questionnaires estimating the subjects’ current status (as a percentage) using a visual analog scale [22] at three time points (before, during, and after the training). Loaded physical exercise was evaluated based on levels of serum enzyme proteins derived from muscles (creatine phosphokinase [CPK] and lactate dehydrogenase [LDH]). Using a wearable watch-type wrist bundled sensor with a finger sensor [23], the quality and quantity of sleep during an entire night were evaluated before and during the training.

## Materials and Methods

### Subjects

Members of the Japan Self Defense Force 1^st^ Airborne Brigade who attended the 9-week ranger training program were recruited to the study. All participants provided written informed consent to participate in the present study. The participants were confirmed to have no serious physical diseases and previous psychiatric illnesses in their annual routine physical examination and the examination for the selection to participate in the present ranger training. The participants needed to have enough physical exercise capabilities on the ground and in the water that were monitored at the daily trainings. Members were from Camp Narashino, and the training was executed in the Narashino camp and Hota exercise field. The contents of the training were scheduled well and the intensity of the daily physical exercise was constant during the training period.

All participants were male and their average age was 26.6±3.1 years old (n = 61). The ethical board of the National Defense Medical College approved the present study. Regular daily checkups of the health of the participants were performed by the attending nurses and emergency medical technicians, and the visiting physicians checked their general health at the end of both the 4-wk base training and the 4-wk field training.

### Schedule

**Figure 1:**
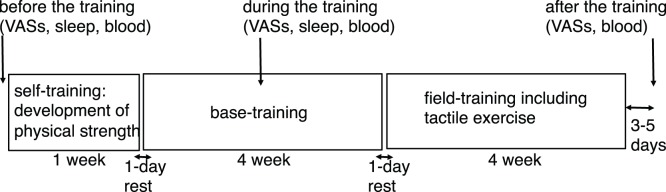
Schedule of VAS questionnaire estimation, sleep evaluations, and blood examinations. VAS questionnaires and blood samples were collected at three time points; before, during, and after training. Sleep was evaluated at two time points; before and during training. The time point of before the training was two days before initiation of training, during training was 2 weeks after the start of base training, and after the training was 3–5 days after cessation of the training period.

Questionnaires and blood samples were collected at three time points: before, during (three weeks after the beginning of training), and 3–5 days after completing training. Movements recorded from the wrist and pulses from a finger during sleep were assessed at two time points: 1–2 days before training and during training (Figure 1).

### Blood Samples

All blood samples were collected at 08∶30 after breakfast, which was at 07∶00. Blood (7 ml) was collected from the forearm vein into EDTA-2Na-containing vacuum tubes (VP-NA070K, Terumo, Tokyo, Japan), and 4 ml was collected into plain vacuum tubes (VP-AS054K, TERMO). Blood in the EDTA-containing tubes were centrifuged at 3000 *g* for 15 min at 4°C. Plasma was collected and stored at −80°C until used for analyses. Blood in the plain tubes was incubated for 30 min at room temperature and centrifuged at 3000 *g* for 5 min. The supernatant serum was collected and stored at −80°C until used for analyses.

### Questionnaire

We used a visual analog scale (VAS) to assess stress and fatigue levels. The visual analog scale measures subjective perception as length. The line segment is 100 mm long. In the fatigue scale, the left edge (0) represents the best condition without any fatigue, and the right edge (100) is the worst condition in a life full of fatigue and in situations in which he or she cannot do anything. In the stress scale, the left edge (0) represents the best condition without stress, and the right edge (100) is the worst condition in a life with extreme stress. Participants were requested to indicate their present fatigue and stress status on the lines.

### Sleep

In the training camp, room lights were to be shut off between 23∶00 and 06∶30. Wrist motion and pulse wave intervals were measured using a wearable watch-type wrist sensor and a finger sensor (NEM-T1, Toshiba, Japan). The watch type sensor (50×60×13 mm in size and 35 g in weight with a rechargeable battery) contains a three-dimensional accelerometer, and the finger sensor (5 g in weight) bundled around the root of the index finger contains a sphygmograph. Sleep duration and autonomic nervous system activity were analyzed using PC software (Nemrism, Toshiba). The accelerometer detects motion that is larger than 0.01 G in the scalar of 3-axes, and the data indicates the sleep/wake rhythm [24], [25]. The sphygmograph detects the pulse wave. The pulse peak interval (PPI) variation measured by the sphygmograph reflects autonomic nervous activity [26]. Fast Fourier Transformation was adopted to analyze the frequency of variability of PPI, and the high frequency (HF, representing parasympathetic nervous system activity) index and low frequency (LF, representing both parasympathetic and sympathetic nervous system activity) index was determined at every time point during sleep. The PC-guided algorithm distinguished sleep stages into wakefulness, rapid eye movement (REM), shallow (stages 1 and 2) non-rapid eye movement (NREM), and deep (stages 3 and 4) NREM sleep periods according to both autonomic nervous system and motor activity. The LF/HF ratio was calculated to represent sympathetic nervous activity, and the duration of NREM deep sleep (slow wave sleep) with particularly low LF/HF ratios was determined. In the present study, data regarding the total sleep period (min), deep sleep period (min) that corresponds to NREM slow-wave sleep, and the HF superior time (min) that corresponds to NREM sleep, are presented.

### Serum Protein Level Measurements

Serum creatine phosphokinase (CPK) and lactate dehydrogenase (LDH) measurements were performed using an automatic analyzer (Labospect-008, Hitachi, Tokyo, Japan).

### Plasma BDNF and VEGF Measurements

Plasma BDNF and VEGF concentrations were measured using the Bioplex 200 suspension system (Bio-Rad Laboratories Inc., Hercules, CA, USA) according to the manufacturer’s enzyme-linked immunosorbent assay protocol, and using kits to measure BDNF (LHC7071, Invitrogen, USA) and VEGF (M50-0KCAF0Y, Bio-Rad).

### Statistical Analyses

All data are presented as the mean ± the standard deviation of the mean. Statistical significance was determined using one-way repeated analysis of variance (ANOVA) followed by Tukey’s multiple comparisons test. Two tailed paired t-tests were used when two time points in the same individual were compared. For analyses of correlations between ΔBDNF and various parameters, and between ΔVEGF and various parameters, Pearson’s correlation coefficients were examined.

## Results

Sixty-one subjects (26.6±3.1 years old) gave written informed consent to participate in the study. During the full training course, 6 subjects dropped out of the training mission due to infectious or orthopedic muscle diseases. No signs of emerging mental problems or psychiatric illnesses during the training mission were subjectively or objectively reported. A total of 59 subjects (26.6±3.2 years old) completed the VAS questionnaires, provided blood samples, and participated in the sleep record during the training, and a total of 55 subjects (26.6±3.0 years old) completed the VAS questionnaires and provided blood samples after the training. Among 55 subjects the amount of plasma from 3 subjects at the time point of before the training period was insufficient due to inappropriate sealing of the tube and their plasma BDNF concentrations could not be analyzed. Therefore, data from 52 subjects (26.6±3.1 years old) were analyzed for plasma BDNF and data from 55 subjects were analyzed for plasma VEGF concentrations. The amount of serum from one subject was insufficient to be analyzed, and data from 54 subjects (26.6±3.1 years old) were analyzed for serum CPK and LDH concentrations. Sleep analyses were performed before and during the training, and the paired records of the total sleep were obtained from 55 subjects (26.7±3.2 years old). Actually 59 subjects participated at the time point of recording of sleep, 4 subjects failed to switch on the sensor before going to sleep. Complete sleep records using the wearable watch-type sensor were obtained from 34 (26.1±3.0 years old) of the 55 subjects. In 21 subjects, the record was not thoroughly completed because unexpected movement during sleep resulted in insufficient contact of the finger sensor to the index finger. Total sleep time data from 55 subjects and autonomic parameters (deep sleep time and HF superior time) data from 34 subjects were analyzed.

### Questionnaires

**Figure 2:**
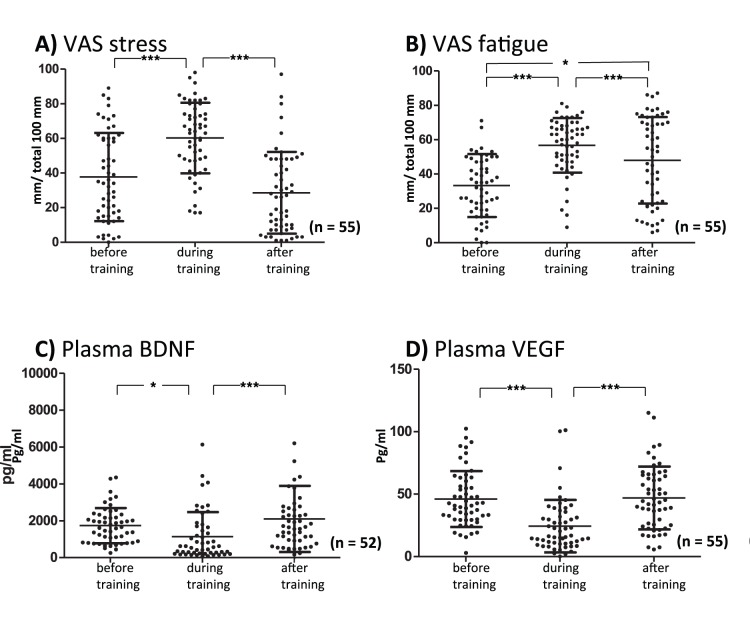
Stress (A; F_2,54_ = 34.8) and fatigue (B; F_2,54_ = 21.5) rated on the visual analog scale (VAS) are plotted before, during, and after training. The results of plasma brain-derived neurotrophic factor (BDNF, C; F_2,51_ = 7.8) and vascular endothelial growth factor (VEGF, D; F_2,54_ = 20.7) are plotted before, during, and after training. Significant differences between groups are indicated as *p<0.05, **p<0.01, and ***p<0.001 using repeated one-way ANOVA followed by post hoc Tukey’s multiple comparison tests.

As shown in Figure 2A, the stress scores on the VAS (before training: 37.7±25.4 mm) significantly increased during the training (60.2±20.4 mm, *p*<0.001). After training, stress scores significantly decreased to baseline (28.6±23.6 mm, *p*<0.001). The F value of the repeated measures ANOVA in the VAS stress scores was F_2.54_ = 34.8. As shown in Figure 2B, the fatigue scores in the VAS (before training: 33.3±18.3 mm) significantly increased during the training (56.7±15.9 mm, *p*<0.001). After training, fatigue scores significantly decreased (48.0±25.1 mm, *p*<0.001) but still remained higher than before the training (*p*<0.05). The F value of the repeated measures ANOVA in the VAS fatigue scores was F_2.54_ = 21.5.

### Escaping Enzymes from Muscles

**Figure 3:**
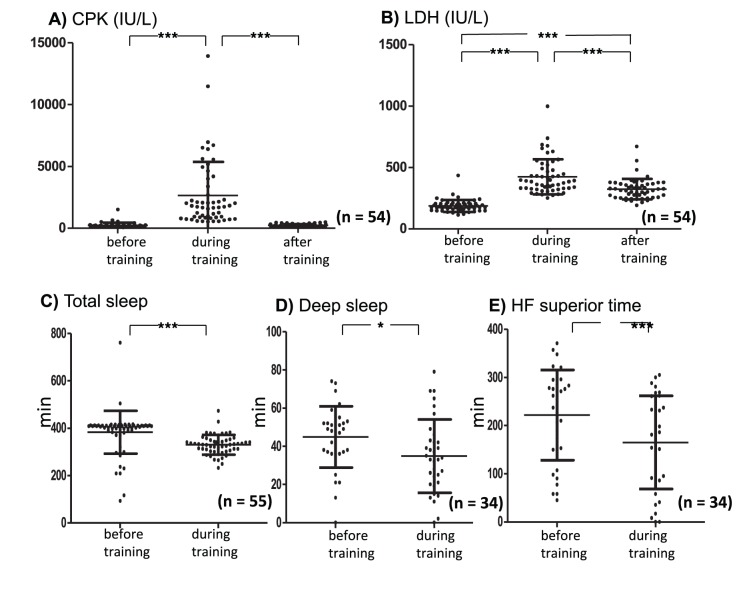
Plasma creatine phosphokinase (CPK; A, F_2,53_ = 42.8) and lactate dehydrogenase (LDH; B, F_2,53_ = 92.1) are plotted before, during, and after training. Total sleep time (C), deep sleep time (D), and HF superior time (E) are plotted before and during training. Significant differences between the two groups in C, D, and E were evaluated using two-tailed paired Student’s t-tests. Significant differences between groups are indicated as *p<0.05, **p<0.01, and ***p<0.001.

As shown in Figure 3A, serum CPK concentrations (before training: 221±202 IU/L) significantly increased above normal limits during the training (2521±2629 IU/L; *p*<0.001). After the training, CPK concentrations were normalized (210±107 IU/L). F value of the repeated measures ANOVA in serum CPK was F_2. 53_ = 42.8. As shown in Figure 3B, serum LDH concentrations (185±47 IU/L) significantly increased above normal limits during training (421±137 IU/L), and despite significantly decreasing after training (321±84 IU/L; *p*<0.001) remained significantly higher than before training (*p*<0.001). F value of the repeated measures ANOVA in serum LDH was F_2. 53_ = 92.1.

### Plasma BDNF and VEGF

As shown in Figure 2C, plasma BDNF levels (before training: 1738±948 pg/ml) significantly decreased during training (1130±1314 pg/ml, *p*<0.05). After training, they returned to baseline levels (2114±1777 pg/ml). The F value of the repeated measures ANOVA in plasma BDNF was F_2. 51_ = 7.8. Plasma VEGF levels (before training: 46.0±22.5 pg/ml) significantly decreased during training (24.3±21.0 pg/ml, p<0.001), but after training returned to baseline levels (46.9±25.2 pg/ml, p<0.001; Fig. 2D). The F value of the repeated measures ANOVA in plasma VEGF was F_2. 54_ = 20.7.

### Sleep

We assessed total sleep time data that were measured by an accelerometer for the 55 subjects. Total sleep time (before training: 380±93 min) significantly decreased during training (330±41 min, p<0.001; t = 3.92, df = 54). We assessed deep sleep time and HF superior time data from 34 subjects. Deep sleep time (before training: 44.8±16.0 min) in the NREM sleep significantly decreased during training (34.9±19.2 min, p<0.05; t = 2.28, df = 33). HF superior time (before training: 221±93 min) significantly decreased during training (165±96 min, p<0.001; t = 4.10, df = 33).

### Relationship between ΔBDNF and Various Parameters

**Figure 4:**
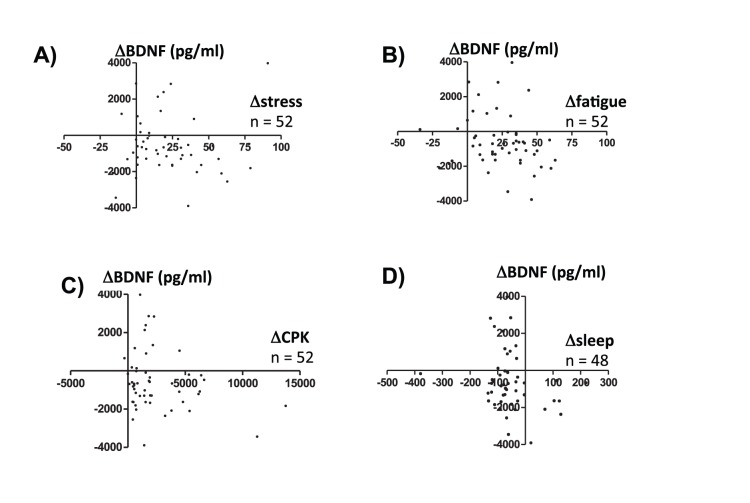
The relationship between ΔBDNF and Δstress (A), ΔBDNF and Δfatigue (B), ΔBDNF and ΔCPK (C), and ΔBDNF and Δsleep (D) are plotted. The values before training were subtracted from the values during the training and are expressed as **Δ**values. No significant correlations were found in the four combinations.

In Figure 4, the relationships between ΔBDNF and Δstress measured in the subjective VAS questionnaire (Fig. 4A; R = 0.023, p = 0.87), ΔBDNF and Δfatigue measured in the subjective VAS questionnaire (Fig. 4B; R = −0.189, p = 0.18), ΔBDNF and Δserum CPK (Fig. 4C; R = −0.246, p = 0.08), and ΔBDNF and Δtotal sleep (Fig. 4D; R = −0.222, p = 0.13) are plotted. No significant correlations were noted in any of these four comparisons. A total of 59 subjects completed the VAS questionnaires, provided blood samples, and participated in the sleep record during the training. Among 59 subjects the amount of plasma from 7 subjects was insufficient due to inappropriate sealing of the tube and their plasma BDNF concentrations could not be analyzed.

### Relationship between ΔVEGF and Various Parameters

**Figure 5:**
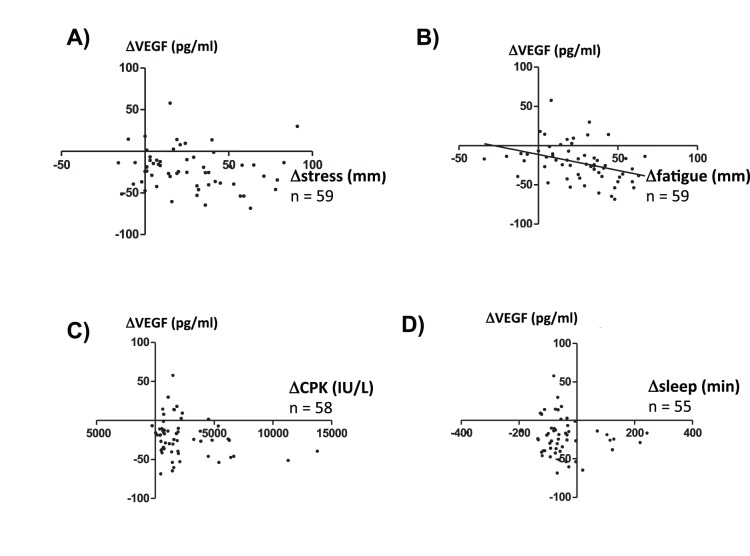
The relationship between ΔVEGF and Δstress (A), ΔVEGF and Δfatigue (B), ΔVEGF and ΔCPK (C), and ΔVEGF and Δsleep (D) are plotted. The values before training were subtracted from the values during training and were expressed as Δvalues. A significant negative correlation was noted between ΔVEGF and Δfatigue (B; R = −0.375, p = 0.003).

In Figure 5, the relationship between ΔVEGF and Δstress measured in the subjective VAS questionnaire (Fig. 5A; R = −0.150, p = 0.26), ΔVEGF and Δfatigue measured in the subjective VAS questionnaire (Fig. 5B; R = −0.375, p = 0.003), ΔVEGF and Δserum CPK (Fig. 5C; R = −0.243, p = 0.07), and ΔVEGF and Δtotal sleep (Fig. 5D; R = −0.104, p = 0.45) are plotted. A significant negative correlation was observed between ΔVEGF and *Δ*fatigue; other three comparisons were non-significant.

## Discussion

The current study revealed a significant decrease in plasma BDNF concentrations 3 weeks after beginning military training. Plasma BDNF returned to pre-training levels 3 to 5 days after completion of the training period. Decreased plasma BDNF concentrations in patients with depression [6], [27] and its positive correlation with cerebrospinal fluid BDNF concentration [28] have previously been demonstrated. Decreased plasma BDNF concentrations in healthy volunteers with psychosocial stress have also been shown [7]. Plasma BDNF concentration more accurately reflects brain BDNF levels compared to serum BDNF because serum BDNF is predominantly derived from clotted peripheral platelets [8]. Serum BDNF, however, has been shown to be decreased by depression [29] and psychosocial stress [30]. Loaded mental stress in the current study was subjectively proven by the significant increases in VAS-stress and VAS-fatigue questionnaire scores during the mission period. Our data suggested that a decrease in plasma BDNF could be an objective stress biomarker candidate in military mission situations.

Blood VEGF concentrations in depressed patients and its changes upon antidepressant treatment have been reported less often compared to the abundant number of BDNF studies. Although a negative correlation between HAMD score and serum VEGF concentration [15] and the observation that higher plasma VEGF levels predicted non-responders in antidepressant treatment [31] have been described, conflicting results [17], [32] were also reported. The effects of stress on human blood VEGF concentrations have never been systematically studied. In addition, little is known about the transport of VEGF protein through the blood-brain barrier. However, down-regulation of hippocampal VEGF expression induced by stress [33], and up-regulation of hippocampal VEGF expression by electroconvulsive seizure [34] and antidepressant [35] treatments have been demonstrated in experimental animals. The significant decrease in plasma VEGF levels that we observed during military training raises the possibility that plasma VEGF can be another stress biomarker candidate.

Muscle exercise and exhaustion during the training are well demonstrated by elevated levels of CPK and LDH [36], and partially demonstrated by elevated levels of VAS-fatigue. The reported activity half-lives of serum CPK-MM and CPK-MB isozymes from muscle tissues are approximately 15 and 12 hours, respectively [37]. The half-lives of various LDH isozymes in muscles are longer, and ranged from 1 to 31 days [38]. Therefore, the serum LDH concentration after training was still significantly higher than the basal level before training due to the longer half-lives of LDH isozymes. The BDNF expressions in the brain [10], in the serum [39], and in the plasma [11] are reported to be increased by physical exercise. The loaded physical exercise in the present study did not affect the significant decrease in plasma BDNF.

Deterioration in the quantity and quality of sleep during training was demonstrated using a non-invasive small watch-type sensor that records wrist motion and heart rate variability. The LF/HF variable represents sympathetic nervous system activation that is increased in REM sleep and decreased in NREM sleep, particularly in deep sleep [26]. This algorithm has been validated by comparing it with the hypnogram data from polysomnography [40]. Although single or multiple sleep deprivation therapies have been reported to increase serum BDNF concentration during treatment for depression [13], the decreased quantity and quality of sleep during the present mission did not affect the significant decrease in plasma BDNF in the current study. A recent pilot study reported significant increases in plasma VEGF after 39 hours of sleep deprivation in major depressive disorder patients [14]. The plasma VEGF concentration decreased in the present mission despite limited amounts and quality of sleep.

The observed decrease in plasma BDNF concentration did not correlate with subjectively measured Δstress-VAS questionnaire answers, Δfatigue-VAS questionnaire answers, Δserum CPK, or Δsleep time. The stress resilience and vulnerability in each individual, as well as their physical exercise and quantity of sleep, are likely related to BDNF expression. We consider it reasonable that we cannot obtain significant correlations between Δplasma BDNF concentration and each parameter measured in this present study. Many parameters contribute to the regulation of plasma BDNF concentration, and no single parameter measured in the current study was sufficient to regulate it. In the case of ΔVEGF concentration, it negatively correlated with Δfatigue-VAS questionnaire answers but not with Δstress-VAS questionnaire answers. There is the possibility that fatigue indicates severe physical and mental exhaustion in participants of this military training. Although speculative, decreased VEGF correlates more with severe mental and physical stress than BDNF. To further support this hypothesis, more studies that evaluate plasma concentrations of neurotrophins in occupational environments are needed. In future studies, elucidation of the relationship between lower plasma neurotrophin levels and the vulnerability to have psychiatric disorders, including depression and posttraumatic stress disorder, is needed.

There were several limitations in this study. First, the participants in the present study were all young Japanese males who had high exercise capabilities, and thus, these results are not relevant to the general population in military missions. Secondly, subjective stress and fatigue were not studied in the most rigorous form in the present study. This was because we had to avoid extra burdens due to the use of complicated questionnaires during the training. Although the 0 and 100 points were defined, the mid point was not defined in the questionnaires.

The present study demonstrated subjectively elevated stress and fatigue, objective increases in serum muscle enzymes, and decreased quantity and quality of sleep during a military training. The most important finding was decreased plasma BDNF and VEGF concentrations during the mission, suggesting the possibility of plasma BDNF and VEGF as biological stress markers in various actual situations, including physical exercise and decreased sleep situations.
